# Supplementary material for: Impact of Theaflavins-Enriched Tea Leaf Extract TY-1 against Surrogate Viruses of Human Norovirus: In Vitro Virucidal Study
Source: Pathogens. 2022 May 2;11(5):533. doi: 10.3390/pathogens11050533 (PMC9147082; doi:10.3390/pathogens11050533)
Supplement: Supplementary file 1 [file pathogens-11-00533-s001.zip › pathogens-1621859-supplementary.pdf]

Table S1. Chemical constituents of 10 mg of TY-1 powder [33,34].

**Item**		**Value**
**Total theaflavins (TFs) (0.165 mg)**	Theaflavin (TF1)	0.112 mg
	Theaflavin-3-gallate (TF2A)	0.029 mg
	Theaflavin-3'-gallate (TF2B)	0.014 mg
	Theaflavin-3,3'-digallate (TF3)	0.010 mg
**Total catechin (0.067 mg)**	Epicatechin (EC)	0.035 mg
	Epigallocatechin (EGC)	0.005 mg
	Epigallocatechin gallate (EGCG)	0.025 mg
	Epicatechin gallate (ECG)	0.002 mg
**Caffeine**		0.180 mg
**Theanine**		0.130 mg
**Gallic acid**		0.104 mg
**Total polyphenol**		1.600 mg
**Dietary fiber**		0.440 mg
**Dextrin**		5.000 mg

Table S2. The sequence of primers targeting FCV VP1 gene and the nonstructural polyprotein gene of the MNV used in this study and each PCR condition.

Primers name	Primer sequences	RT-PCR condition
**FCV-Primer set (264 bp)**	Fwd: 5'-TCCACACTAGCGTCAACTGG-3'<br>Rev: 5'-GACGAGCGTCAAACAGAACA-3'	95 °C for 5 min<br>↓<br>[95 °C for 30 sec,<br>49 °C for 30 sec,<br>72 °C for 1 min]<br>x 22 times<br>↓<br>72 °C for 10 min
**MNV-Primer set (549 bp)**	Fwd: 5'-GCCCACTGGATTCTGACTCT-3'<br>Rev: 5'-GGTCTCAGCATCCATTGTTCC-3'	95 °C for 5 min<br>↓<br>[95 °C for 30 sec,<br>56 °C for 30 sec,<br>72 °C for 1min]<br>x 35 times<br>↓<br>72 °C for 10 min

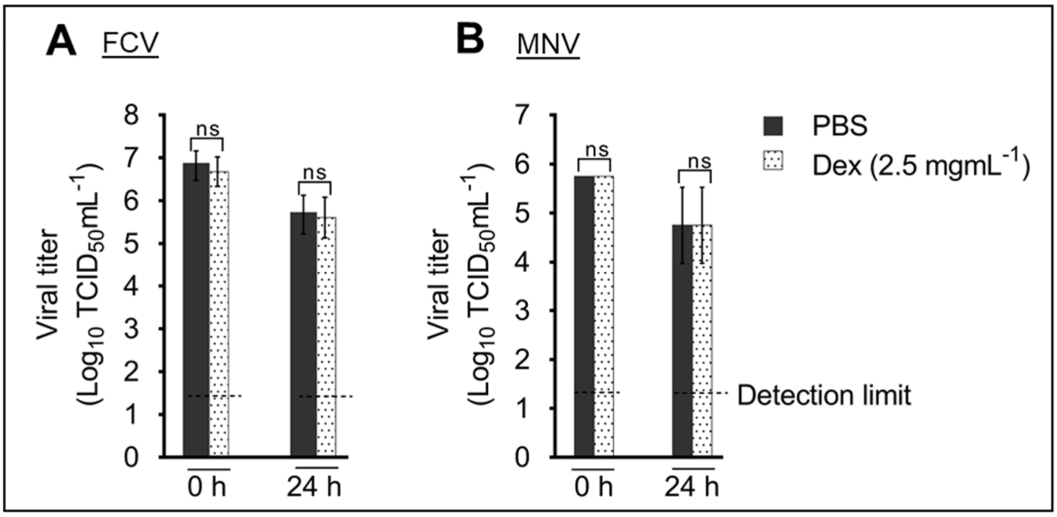

Figure S1. Comparison of the viral titers of viruses treated with PBS and Dex. **(A, B)** The FCV **(A)** or MNV **(B)** solution was mixed with PBS or Dex (final dosage: 2.5 $mgmL^{-1}$). Then, the mixtures were incubated at 25°C for 0 h or 24 h. The data were expressed as mean ± SD (n ≥ 6 per group). The Student's *t*-test was used to analyze the statistical significance of the differences between the PBS and the Dex groups; ns: not significant.

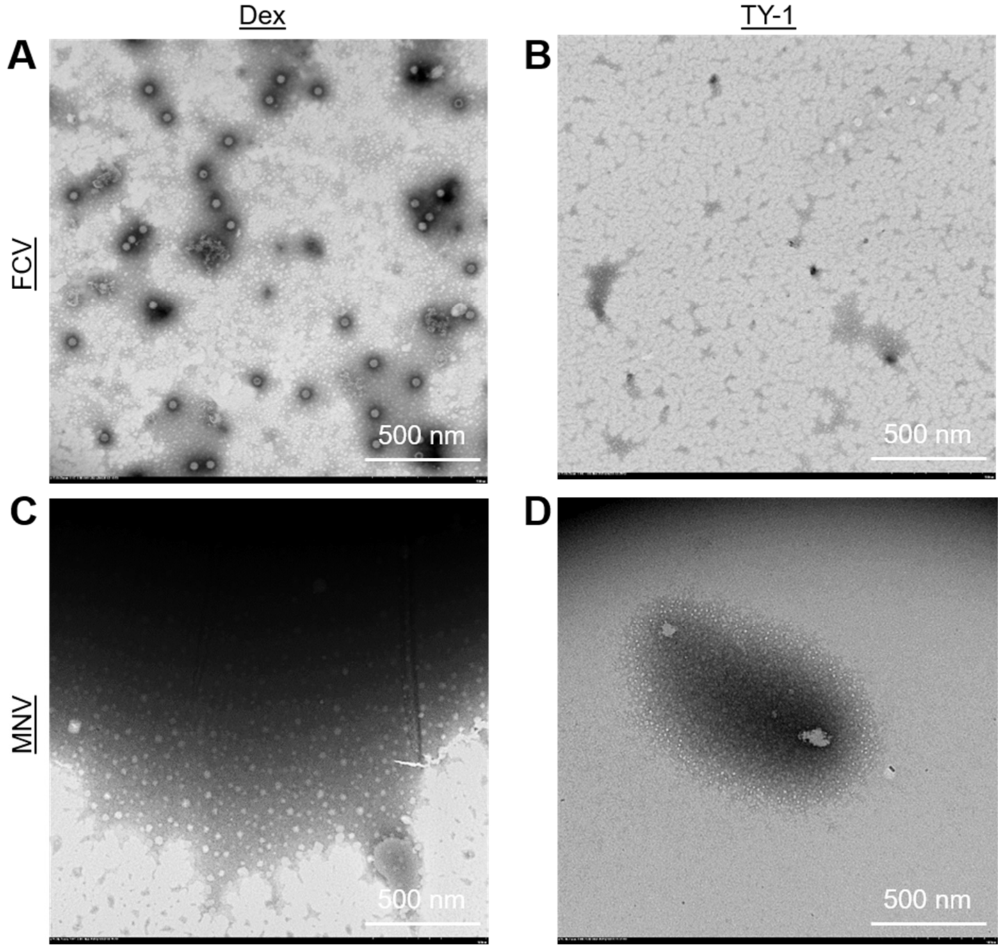

Figure S2. TEM images of Dex- or TY-1-treated FCV and MNV virions under lower magnification field of view. **(A–D)** A solution with purified FCV or MNV was mixed with Dex (final dosage: 2.5 mgmL^{-1}) **(A, C)** or TY-1 (5.0 mgmL^{-1}) **(B, D)** and incubated at 25°C for 6 h. Then, the virions were observed using TEM. The results were descriptive images of Dex- and TY-1-treated viral particles.
